# The Ratio of Hemoglobin to Red Cell Distribution Width: A Strong Predictor of Clinical Outcome in Patients with Heart Failure

**DOI:** 10.3390/jcm11030886

**Published:** 2022-02-08

**Authors:** Eldad Rahamim, Donna R. Zwas, Andre Keren, Gabby Elbaz-Greener, Mahsati Ibrahimli, Offer Amir, Israel Gotsman

**Affiliations:** 1 Heart Institute, Hadassah Medical Center, Faculty of Medicine, Hebrew University of Jerusalem, Jerusalem 91120, Israel; eldad.rahamim@gmail.com (E.R.); donnaz1818@gmail.com (D.R.Z.); kerena@mail.huji.ac.il (A.K.); gabbyelbaz@yahoo.com (G.E.-G.); mehseti.ibrahimli@gmail.com (M.I.); oamir@hadassah.org.il (O.A.); 2 Heart Failure Center, Clalit Health Services, Tel Aviv 16250, Israel

**Keywords:** hemoglobin, red cell distribution width, heart failure, outcome, prognosis

## Abstract

Background: Hemoglobin (Hb) is a standard and widely available clinical parameter that predicts clinical outcomes in heart failure (HF) patients. Red cell distribution width (RDW) is also a routinely measured clinical parameter that is predictive of clinical outcomes in HF. The ratio between Hb and RDW has yet to be evaluated in HF. Methods: We evaluated the predictive value of the Hb/RDW ratio on clinical outcomes in patients with HF. All patients diagnosed with chronic HF at a health maintenance organization were evaluated for Hb/RDW ratio and followed for cardiac-related hospitalizations and death. Results: The study cohort included 6888 HF patients. The mean Hb/RDW ratio was 0.85 ± 0.18; median was 0.85 (interquartile range 0.72–0.98). Patients with a lower Hb/RDW ratio were more likely to be women and had more comorbidities. The overall two year-mortality rate was 23.2%. Decreasing quantiles of the Hb/RDW ratio were associated with reduced survival rates and reduced event-free survival from death or cardiovascular-hospitalizations. Multivariable Cox regression analysis after adjustment for significant predictors demonstrated that low Hb/RDW ratio was a significant predictor of mortality, with a graded increased risk as Hb/RDW ratio decreased. Lower Hb/RDW ratio was also a significant independent predictor of the combined endpoint of death or cardiovascular hospitalizations. A sensitivity analysis evaluating Hb/RDW ratio as a continuous parameter using restricted cubic splines demonstrated a continuous increase in the mortality risk with decreasing Hb/RDW ratio, *p* < 0.0001 for the linear model. Conclusions: Hb/RDW ratio is a significant prognostic tool for predicting HF mortality and cardiovascular hospitalizations.

## 1. Introduction

Heart failure (HF) has emerged as a major epidemic and is a significant public health burden that is associated with considerable morbidity and mortality [1]. Prediction of clinical outcomes is paramount in HF, and there are numerous clinical parameters that are used to predict clinical outcomes in heart failure. Hemoglobin (Hb) is a standard and widely available clinical parameter that has a significant impact on outcomes in heart failure patients [2] as anemia reflects the nutritional, inflammatory, renal and general status of HF patients [3]. Red cell distribution width (RDW) is another hematological parameter that measures the degree of anisocytosis in red blood cells. RDW is the coefficient of variance of the size of red blood cells and is a useful parameter in the classification of anemia [4]. Anisocytosis reflects a major deregulation of red blood cell homeostasis, with impaired erythropoiesis and red blood cell survival. These impairments are attributed to shortening of telomer length, oxidative stress, poor nutrition, dyslipidemia, hypertension, and inflammation [5]. Increased RDW therefore represents numerous biological processes including inflammation, aging, oxidative stress, nutritional deficiencies, and impaired renal function. Multiple studies have established this parameter as a predictor of cardiovascular outcomes [6,7,8]. In a prospective study, increased RDW was found to be a major risk factor for all-cause mortality and was associated with increased risk of death from cardiovascular disease [9]. RDW has been shown to have significant predictive valve in chronic [10,11] as well as acute HF [12], with higher levels of anisocytosis predicting 1-year mortality in acute HF.

As these two parameters are readily available with the standard complete blood cell count and both are significant predictors of outcome in HF, the ratio of hemoglobin to RDW (Hb/RDW ratio) should provide incremental clinical prediction as it reflects and encompasses a wide range of clinical characteristics of these patients. To our knowledge, there is no published data regarding the clinical significance of the Hb/RDW ratio in HF. The purpose of the present study was to evaluate the Hb/RDW ratio as a predictor of cardiovascular outcomes in a large real-world cohort of patients with chronic HF.

## 2. Methods

Clalit Health Services is the largest health maintenance organization (HMO) in Israel. It has a central computerized database in which all members have a complete digital record. The database includes demographic data, comprehensive clinical data, diagnoses, and laboratory data undertaken in a single centralized laboratory of the HMO. We identified and retrieved electronically from the computerized database all members with a clinical diagnosis of HF as coded by the database in the Jerusalem district. Data were retrieved from January 2017. Patients were followed for clinical events, including cardiovascular hospitalizations and death, from January 2017 until January 2019. Overall, 6888 patients had a diagnosis of HF. Determination of the type of HF, HF with reduced ejection fraction (HFrEF) and HF with preserved ejection fraction (HFpEF) was based on a documented specific diagnosis in the database and was available in 67% of the patients. The diagnosis of the remaining patients was ‘Heart failure, unspecified’. All hospitalizations in cardiac and internal medicine departments, including cardiac and internal intensive care units, were retrieved and analyzed. Data on mortality was retrieved from the National Census Bureau. The Institutional Committee for Human Studies of Clalit Health Services approved the study protocol. 

Biochemical analyses were performed at the HMO single centralized core laboratory with routine standardized methodologies on fresh samples of blood obtained after an overnight fast. Glucose levels were measured in plasma, and all other biochemical analyses were performed on serum. Urinary albumin to creatinine ratio (μg/mg) was measured from a spot morning urine sample. Natriuretic peptides are not routinely performed in Israel and were not available for analysis. The laboratory is authorized to perform tests according to the international quality standard ISO-9001. 

SPSS version 17.0 for Windows (SPSS Inc., Chicago, IL, USA) and R Statistical Software version 3.0.1 for Windows (R Development Core Team) were used for the analyses. Data on continuous variables is presented by median and interquartile ranges as they were not distributed normally. Comparison of the clinical characteristics was performed using the Kruskal–Wallis test for continuous variables and the Chi-Square Test for categorical variables. Clinical predictors were transformed where appropriate. Log_10_ was used for logarithmic transformations except for estimated glomerular filtration rate (eGFR) that a square root transformation was used. Follow-up time was calculated using a Kaplan–Meier estimate of potential follow-up. Kaplan–Meier curves, with the log-rank test, were used to compare survival according to Hb/RDW ratio. Multivariate Cox proportional hazards regression analysis was used to evaluate independent variables that determined survival. Parameters included in the multivariate Cox regression analysis incorporated relevant parameters that were significant on univariable analysis with the addition of significant drug therapy in separate models. Restricted cubic spline multivariable cox regression analysis was performed to evaluate the relationship between Hb/RDW ratio as a continuous parameter and mortality. Proportionality assumptions of the Cox regression models were evaluated by log–log survival curves and with the use of Schoenfeld residuals. An evaluation of the existence of confounding or interactive effects was made between variables and their possible collinearity. Receiver operating characteristic (ROC) curves were constructed for the predictive models of mortality during follow-up. Area under the curve (AUC) was used in order to assess the performance of the models. A *p*-value of <0.05 was considered statistically significant.

## 3. Results

### 3.1. Clinical Parameters

**Figure 1:**
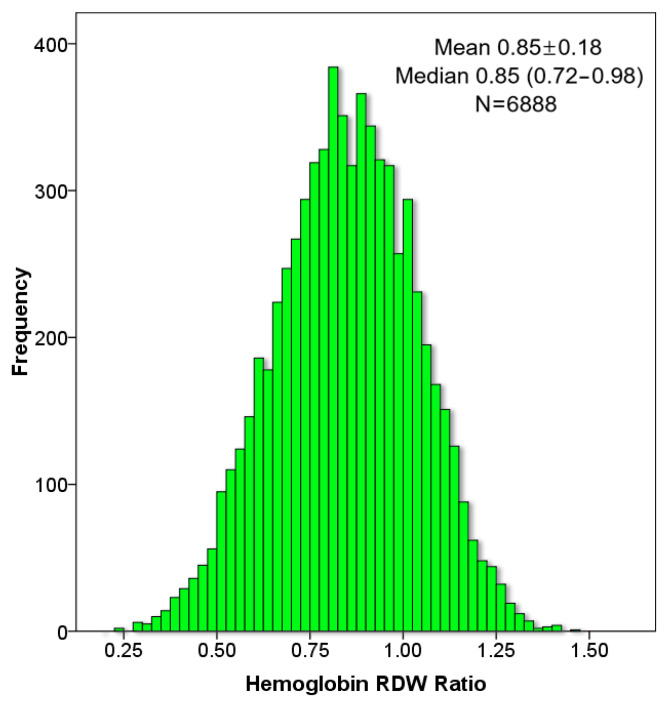
Distribution of the Hb/RDW ratio in the HF cohort.

The study cohort included 6888 HF patients. Figure 1 demonstrates the distribution of the Hb/RDW ratio in the HF cohort. The mean Hb/RDW ratio was 0.85 ± 0.18; and median was 0.85 (interquartile range 0.72–0.98). We divided the cohort into six quantiles based on the Hb/RDW ratio <0.25, 0.25–0.49, 0.5–0.74, 0.75–0.99, 1–1.24, >1.25. The characteristics of the patients stratified according to the Hb/RDW ratio quantiles are presented in Table 1. Patients with a lower Hb/RDW ratio were more likely to be women, and had a higher number of comorbidities and a higher Charlson comorbidity Score. Patients in the highest (6th) quantile were younger than patients in other quantiles. Lower quantiles of Hb/RDW ratio were associated with more advanced NYHA class and with HFpEF. Other predictors of a worse outcome in HF were also associated with a lower Hb/RDW ratio including higher urea, lower eGFR and albumin, as well as lower hemoglobin, iron and transferrin saturation. A lower Hb/RDW ratio was associated with higher C-reactive protein levels. Lower Hb/RDW ratio was associated with lower prescription rates of ACE-I/ARNI, beta-blockers, aspirin, thiazides, and amiodarone, but with higher prescription rates of furosemide and digoxin.

### 3.2. Hb/RDW Ratio and Clinical Outcomes

**Figure 2:**
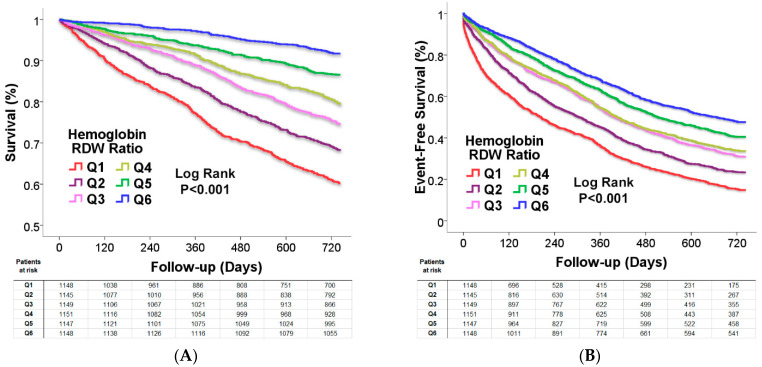
(**A**) Kaplan–Meier analysis of the Hb/RDW ratio association with survival. (**B**) Kaplan–Meier analysis of the Hb/RDW ratio association with event-free survival from death or cardiovascular-hospitalizations.

The overall two year-mortality rate was 23.2%. The survival rate by Kaplan–Meier analysis demonstrated that a lower Hb/RDW ratio score was significantly and incrementally associated with reduced survival. Survival rates increased with increasing quantiles of the Hb/RDW ratio (60.2 ± 1.4% vs. 68.2 ± 1.4% vs. 74.6 ± 1.3% vs. 79.3 ± 1.2% vs. 86.6 ± 1.0% vs. 91.7 ± 0.8, respectively, *p* < 0.001; Figure 2A). Similarly, the lower Hb/RDW ratio was also directly associated with decreased event-free survival from death or cardiovascular-hospitalizations (14.8 ± 1.0% vs. 23.3 ± 1.3% vs. 30.9 ± 1.4% vs. 33.7 ± 1.4% vs. 40.5 ± 1.5% vs. 47.6 ± 1.5%, respectively, *p* < 0.001; Figure 2B). Multivariable Cox regression analysis after adjustment for significant predictors including comorbidities and laboratory parameters demonstrated that a lower Hb/RDW ratio was a significant predictor of mortality (Table 2). After adjustment for other significant predictors (see Table 2 for predictors included), lower Hb/RDW ratio was associated with an incremental increase in mortality. Inclusion of HF medications in the model demonstrated a similar result (Table 3). Lower Hb/RDW ratio was also a significant independent predictor of the combined endpoint of death or cardiovascular hospitalizations with a graded increased risk with decreasing Hb/RDW ratio (Table 3). Inclusion of iron status (iron deficiency defined as Ferritin <100 ng/mL or Ferritin <300 ng/mL associated with transferrin saturation <20%) in the adjusted model did not change the predictive value of the Hb/RDW ratio; it remained a strong predictor of survival as well as death or cardiovascular hospitalizations.

**Figure 3:**
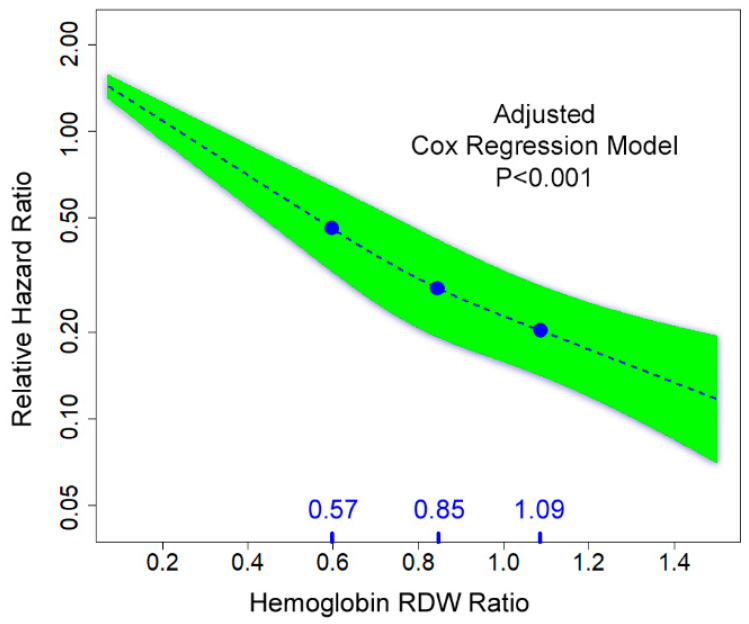
Sensitivity analysis evaluating Hb/RDW ratio as a continuous parameter using restricted cubic splines.

Analyzing the Hb/RDW ratio as a continuous parameter by Cox regression analysis after adjustment for the above predictors and HF medications demonstrated a significant 19% decrease in mortality with each 0.10 unit increase in the Hb/RDW ratio (HR 0.81, 95% CI 0.79–0.84, *p* < 0.0001). A sensitivity analysis evaluating Hb/RDW ratio as a continuous parameter using restricted cubic splines was performed. Knots were allocated at Hb/RDW ratio of 0.57, 0.85, and 1.09. Cox regression analysis demonstrated a direct relationship between lower Hb/RDW ratio and mortality, after adjustment for significant parameters included in Table 2. This analysis demonstrated that there was a continuous increase in the risk with decreasing Hb/RDW ratio (Figure 3), *p* < 0.0001 for the adjusted linear model. 

**Figure 4:**
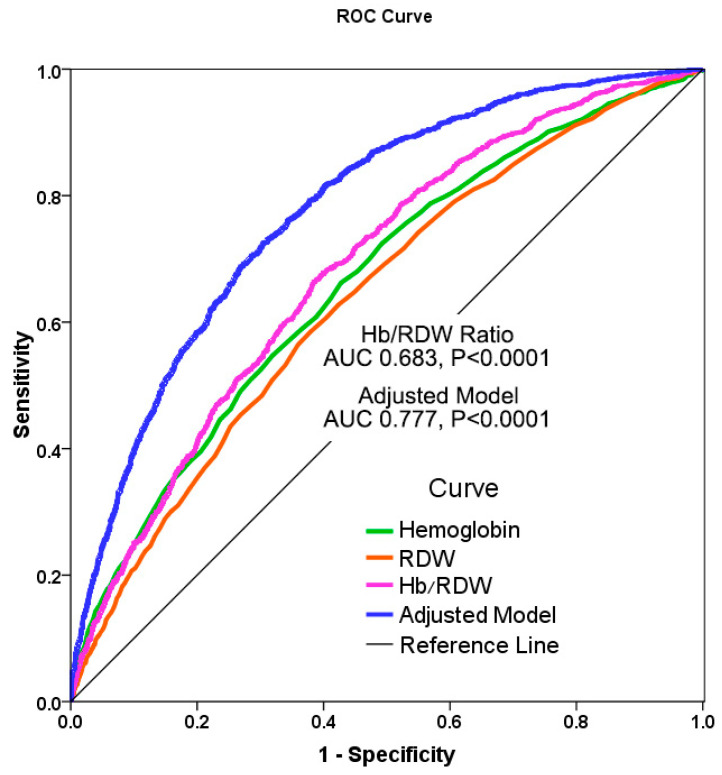
Receiver operator characteristics curve of the Hb/RDW ratio and the adjusted model for mortality in the HF cohort. The Hb/RDW ratio had good predictive value compared to the standard multivariable adjusted model and was better than Hb and RDW. Hb/RDW ratio: AUC = 0.683, *p* < 0.0001; multivariable adjusted model: AUC = 0.777, *p* < 0.0001; Hb: AUC = 0.662, *p* < 0.0001; RDW: AUC = 0.638, *p* < 0.0001.

We also looked at the predictive value of the Hb/RDW ratio as a stand-alone predictor compared to the standard multivariable adjusted model in our cohort, assessing the receiver operator characteristics (ROC) curve for mortality. Although the adjusted model incorporating numerous HF specific parameters including NYHA class was better than the Hb/RDW ratio (AUC = 0.777, *p* < 0.0001), the Hb/RDW ratio had a good predictive value on its own (AUC = 0.683, *p* < 0.0001); Figure 4. Hb alone had a lower predictive value (AUC = 0.662, *p* < 0.0001). A similar result was found analyzing the predictive value using Harrell’s C statistic for predicting survival (C = 0.751, *p* < 0.0001 for the adjusted model; C = 0.660, *p* < 0.0001 for the Hb/RDW ratio).

The predictive value of the Hb/RDW ratio was present over the entire cohort of HF patients including patients with HFrEF and in HFpEF (Supplemental Table S1). In all groups, the Hb/RDW ratio was a significant predictor of mortality and was associated with an incremental increase in mortality by itself and after adjustment for significant predictors. Similarly, lower values of the Hb/RDW ratio were a predictor of the combined endpoint of event-free survival from death or cardiovascular hospitalization.

## 4. Discussion

The present study demonstrates that a low Hb/RDW ratio was a significant predictor of death as well as the combined endpoint of death or cardiovascular hospitalization in a real-world cohort of HF patients. There was a direct incremental relationship between low Hb/RDW ratio and clinical outcomes. The predictive value was present after adjustment for iron status and was present over the whole spectrum of HF types, including HFrEF an HFpEF.

The Hb/RDW ratio is a clinical parameter that incorporates two parameters, hemoglobin and RDW, that are readily available with the standard blood count. Both these parameters have significant prognostic value. Hemoglobin has been shown to be an important clinical predictor of outcome in HF. A low hemoglobin is common in patients with HF, is associated with a poor clinical status and portrays worse outcomes. It is a marker of HF severity as it reflects numerous detrimental factors in HF patients including chronic inflammation, malnutrition, iron deficiency, bleeding, and chronic kidney disease [3]. It is not surprising that a lower hemoglobin represents worse outcomes in HF patients. RDW is another hematological parameter that measures the degree of anisocytosis in red blood cells due to impaired erythropoiesis and red blood cell survival. Increased RDW represents numerous biological processes including inflammation, aging, oxidative stress, nutritional deficiencies, and impaired renal function. This parameter is an established parameter of cardiovascular outcome prediction [6]. A meta-analysis showed that an increase in RDW correlates with an increased risk of all-cause mortality in cardiovascular patients [6]. In heart failure patients, RDW was found to be a strong independent predictor of morbidity and mortality [4,11,13,14,15]. In patients with acute heart failure, higher RDW levels at discharge were associated with a worse long-term outcome, regardless of hemoglobin levels and anemia status [16] A meta-analysis in patients with HF demonstrated that a 1% increase in RDW was associated with a 9.1% increase in the risk of HF hospitalization and a 18.9% increase in the risk of combined adverse events [17].

Several hypothesized mechanisms for the effect of RDW on cardiovascular outcome and severity of HF have been offered. As RDW represents numerous detrimental biological processes it should adversely impact outcome in HF patients. Increased RDW represents abnormal erythrocyte homeostasis and deformed red blood cells leading to disturbed blood flow in the microcirculation [5]. This could potentially contribute to deterioration in cardiovascular diseases and HF patients. Reverse left ventricular remodeling, an important process in HF patients with cardiac resynchronization therapy devices, is decreased in the presence of increased RDW [18]. Inflammation, an important factor in cardiovascular diseases, is associated with higher values of RDW [19]. This may also explain why higher RDW indices are seen in HF patients with more severe disease [20,21].

Based on the above considerations, it is not unexpected that the combination of these parameters as a ratio has significant prognostic utility in HF. The combination represents a spectrum of comorbidities in HF patients. Anisocytosis may suggest additional metabolic stress beyond the parameters that lead to anemia, and these stressors may have greater adverse impact in the setting of anemia. The Hb/RDW should represent and identify patients with the worst clinical status and with the greatest risk of deterioration. This was seen in the present study. Hb/RDW was associated with numerous predictors of worse outcome in HF. Patients with a low Hb/RDW ratio in our study were older, had a higher NYHA functional class, higher rate of comorbidities including diabetes, atrial fibrillation, renal failure, prior stroke and PVD, as well as increased inflammation as represented by C-reactive protein and lower albumin and iron indices. These patients were also prescribed less sympathetic and renin-angiotensin system blockers, but were prescribed more diuretics, suggesting more severe HF patients. All these characteristics portray patients with a poor prognosis. This parameter represents and encompasses a broad spectrum of detrimental characteristics in HF patients and creates a robust prognostic tool to identify HF patient at greater risk for increased morbidity and mortality. 

The strengths of combining RDW and Hb into a prognostic ratio was reported in a meta-analysis suggesting it as a powerful tool for prognostication in cardiovascular diseases [6]. The Hb/RDW ratio has been used as a prognostic factor in elderly patients and in various malignancies. Hb/RDW ratio was found to be a prognostic indicator for frailty in the elderly and was suggested to assist with identifying patients at risk [22]. A significant association was found between Hb/RDW ratio and clinical characteristics and survival outcomes in patients with malignancies [23,24]. This is the first time that this relationship has been described in heart failure patients and it prognostic value. This ratio is able to identify patients with more comorbidities and thus identify patients at highest risk of death and hospitalization. The Hb/RDW ratio is a simple and readily available predictive tool that may help the clinician in risk stratification of heart failure patients. 

## 5. Limitations

Several potential limitations of this study merit consideration. The present study was an observational study. Data regarding clinical parameters and drug therapy was based on a digitized database. Although this database was validated and found to be highly accurate, not all data could be verified. While we tried to adjust for clinically relevant parameters, not all clinical parameters were available, and it is impossible to adjust for all variables that may affect the outcome. Data on natriuretic peptide levels were not available. Data on the specific HF type was not available in all the patients, which may cause bias. In addition, the cohort was community-based, and the findings may not be applicable in more advanced or hospital-based HF cohorts. 

## 6. Conclusions

In conclusion, Hb/RDW ratio is a significant prognostic tool for the prediction of HF mortality and hospitalizations and can be helpful in the risk stratification of HF patients.

## Figures and Tables

**Table 1:** Demographics and clinical characteristics of patients with heart failure according to the hemoglobin RDW ratio.

Variable	Q1 (N = 1148)	Q2 (N = 1145)	Q3 (N = 1149)	Q4 (N = 1151)	Q5 (N = 1147)	Q6 (N = 1148)	Total (N = 6888)	*p* Value
Age (Years)	79 (69–86)	81 (71–87)	81 (71–87)	79 (70–87)	75 (66–84)	68 (57–78)	78 (67–85)	<0.001
Gender (Male)	470 (41)	480 (42)	494 (43)	523 (45)	703 (61)	930 (81)	3600 (52)	<0.001
NYHA Class III/IV	433 (50)	450 (51)	361 (41)	324 (39)	256 (29)	161 (19)	1985 (38)	<0.001
HF Type								
Reduced ejection fraction	260 (23)	278 (24)	296 (26)	306 (27)	356 (31)	368 (32)	1864 (27)	<0.001
Preserved ejection fraction	541 (47)	504 (44)	494 (43)	456 (40)	398 (35)	332 (29)	2725 (40)	
Not-specified	347 (30)	363 (32)	359 (31)	389 (34)	393 (34)	448 (39)	2299 (33)	
Diabetes mellitus	762 (66)	695 (61)	639 (56)	589 (51)	556 (48)	489 (43)	3730 (54)	<0.001
Hypertension	1037 (90)	1026 (90)	1014 (88)	987 (86)	908 (79)	799 (70)	5771 (84)	<0.001
Hyperlipidemia	1032 (90)	1035 (90)	1023 (89)	1043 (91)	1013 (88)	1022 (89)	6168 (90)	0.42
Ischemic Heart Disease	766 (67)	766 (67)	752 (65)	728 (63)	737 (64)	803 (70)	4552 (66)	0.01
Prior Myocardial Infarction	485 (42)	447 (39)	481 (42)	469 (41)	474 (41)	584 (51)	2940 (43)	<0.001
Prior coronary bypass surgery	25 (2)	21 (2)	19 (2)	22 (2)	14 (1)	19 (2)	120 (2)	0.63
Atrial fibrillation	527 (46)	530 (46)	485 (42)	439 (38)	384 (33)	300 (26)	2665 (39)	<0.001
Prior Stroke/ transient ischemic attack	349 (30)	321 (28)	283 (25)	269 (23)	246 (21)	191 (17)	1659 (24)	<0.001
Peripheral vascular disease	221 (19)	213 (19)	178 (15)	175 (15)	145 (13)	110 (10)	1042 (15)	<0.001
Chronic obstructive lung disease	284 (25)	295 (26)	236 (21)	247 (21)	233 (20)	220 (19)	1515 (22)	<0.001
Charlson Score	7.0 (6.0–8.0)	7.0 (6.0–8.0)	7.0 (5.0–8.0)	6.0 (5.0–8.0)	6.0 (4.0–7.0)	5.0 (4.0–6.0)	6.0 (5.0–8.0)	<0.001
Depression	238 (21)	248 (22)	230 (20)	231 (20)	195 (17)	133 (12)	1275 (19)	<0.001
Dementia	191 (17)	182 (16)	212 (18)	200 (17)	115 (10)	87 (8)	987 (14)	<0.001
Dialysis	178 (16)	98 (9)	45 (4)	36 (3)	10 (0.9)	8 (0.7)	375 (5)	<0.001
Malignancy	345 (30)	294 (26)	263 (23)	256 (22)	224 (20)	158 (14)	1540 (22)	<0.001
Body mass index (kg/m^2^)	29 (25–33)	29 (26–34)	29 (26–34)	29 (25–33)	29 (26–33)	28 (25–32)	29 (25–33)	0.006
Pulse (beats per minute)	73 (65–81)	72 (65–80)	72 (64–80)	71 (64–80)	72 (64–80)	72 (64–81)	72 (64–80)	0.007
Systolic blood pressure (mmHg)	126 (116–138)	128 (118–139)	129 (118–140)	129 (117–140)	128 (118–138)	126 (117–137)	128 (118–139)	0.003
Diastolic blood pressure (mmHg)	70 (61–78)	70 (63–78)	70 (63–79)	70 (65–79)	72 (66–80)	74 (68–80)	71 (64–79)	<0.001
Laboratory Data								
Hemoglobin RDW ratio	0.58 (0.51–0.62)	0.72 (0.69–0.74)	0.81 (0.79–0.83)	0.89 (0.87–0.91)	0.98 (0.95–1.00)	1.10 (1.06–1.16)	0.85 (0.72–0.98)	<0.001
Hemoglobin (g/dL)	10.1 (9.3–10.9)	11.4 (10.8–12.1)	12.1 (11.6–12.7)	12.9 (12.4–13.5)	13.8 (13.2–14.4)	15.1 (14.4–15.9)	12.6 (11.3–14.0)	<0.001
Red Cell Distribution Width (%)	17.9 (16.5–19.7)	15.9 (15.1–16.8)	15.1 (14.4–15.8)	14.5 (14.0–15.1)	14.1 (13.6–14.7)	13.6 (13.1–14.0)	14.8 (13.9–16.1)	<0.001
Creatinine (mg/dL)	1.2 (0.9–1.8)	1.1 (0.8–1.6)	1.0 (0.8–1.4)	1.0 (0.8–1.3)	0.9 (0.8–1.2)	0.9 (0.8–1.0)	1.0 (0.8–1.3)	<0.001
Estimated glomerular filtration rate (mL/min per 1.73 m^2^) *	53 (31–76)	57 (38–80)	63 (46–84)	69 (51–89)	77 (58–96)	88 (72–107)	69 (48–92)	<0.001
Urea (mg/dL)	62 (43–96)	57 (40–84)	50 (37–71)	46 (34–61)	41 (32–54)	37 (29–46)	46 (34–66)	<0.001
Urine Albumin/Creatinine ratio	83 (23–284)	54 (19–224)	44 (16–197)	34 (14–147)	26 (9.0–101)	18 (7.0–74)	38 (13–163)	<0.001
Sodium (mEq/L)	140 (137–142)	140 (138–142)	140 (138–142)	140 (138–142)	140 (138–142)	140 (138–142)	140 (138–142)	<0.001
Potassium (mEql/L)	4.6 (4.3–5.1)	4.7 (4.3–5.0)	4.7 (4.3–5.0)	4.6 (4.3–4.9)	4.6 (4.3–4.9)	4.5 (4.3–4.9)	4.6 (4.3–4.9)	<0.001
White blood count (×10^9^/L)	6.9 (5.6–8.7)	7.1 (5.8–8.6)	7.1 (5.9–8.6)	7.3 (6.0–8.8)	7.5 (6.2–9.0)	7.7 (6.4–9.3)	7.3 (6.0–8.8)	<0.001
Glucose (mg/dL)	109 (93–139)	106 (93–136)	106 (93–134)	105 (94–129)	107 (94–131)	106 (95–132)	106 (94–133)	0.33
Hemoglobin A1c (%)	6.2 (5.6–7.2)	6.2 (5.6–7.2)	6.2 (5.6–7.3)	6.1 (5.6–7.0)	6.1 (5.6–7.0)	5.9 (5.5–6.9)	6.1 (5.6–7.1)	<0.001
Uric Acid (mg/dL)	6.7 (5.2–8.4)	6.5 (5.2–8.1)	6.4 (5.0–7.8)	6.1 (5.0–7.3)	6.2 (5.1–7.4)	6.0 (5.0–7.0)	6.3 (5.1–7.6)	<0.001
TSH (mIU/L)	2.5 (1.6–4.0)	2.5 (1.5–3.8)	2.2 (1.5–3.4)	2.3 (1.4–3.3)	2.1 (1.4–3.2)	2.0 (1.4–2.9)	2.2 (1.5–3.4)	<0.001
Iron (µg/dL)	40 (29–56)	48 (37–62)	57 (43–71)	63 (49–79)	69 (54–87)	81 (63–103)	58 (42–77)	<0.001
Transferrin (mg/dL)	232 (188–290)	240 (201–285)	246 (209–283)	251 (216–292)	251 (225–280)	253 (230–284)	245 (210–285)	<0.001
Transferrin Saturation (%)	13 (8.3–20)	15 (10–20)	16 (12–21)	18 (13–23)	20 (15–26)	23 (17–30)	17 (12–23)	<0.001
Ferritin (ng/mL)	91 (31–259)	83 (33–191)	76 (35–165)	73 (39–145)	83 (47–141)	108 (57–190)	84 (39–179)	<0.001
Calcium (mg/dL)	8.9 (8.5–9.3)	9.1 (8.8–9.4)	9.2 (8.9–9.6)	9.3 (9.0–9.6)	9.4 (9.1–9.6)	9.4 (9.2–9.7)	9.2 (8.9–9.6)	<0.001
Phosphorus (mg/dL)	3.6 (3.2–4.1)	3.6 (3.2–4.0)	3.5 (3.2–3.9)	3.5 (3.1–3.9)	3.4 (3.1–3.8)	3.3 (3.0–3.7)	3.5 (3.1–3.9)	<0.001
Triglycerides (mg/dL)	114 (82–157)	117 (86–161)	119 (89–164)	118 (88–169)	120 (90–171)	131 (96–185)	119 (88–168)	<0.001
Low-density lipoprotein (mg/dL)	75 (56–94)	80 (63–101)	84 (66–107)	85 (67–108)	84 (68–108)	87 (68–115)	83 (65–106)	<0.001
Albumin (g/dL)	3.6 (3.3–3.9)	3.7 (3.5–4.0)	3.8 (3.6–4.1)	3.9 (3.7–4.1)	4.0 (3.8–4.2)	4.1 (4.0–4.3)	3.9 (3.6–4.1)	<0.001
C-Reactive Protein (mg/dL)	1.0 (0.4–2.6)	0.8 (0.3–1.9)	0.7 (0.3–1.7)	0.5 (0.2–1.3)	0.5 (0.2–1.3)	0.4 (0.2–0.8)	0.6 (0.3–1.6)	<0.001
Alanine transaminase (IU)	13 (9.0–19)	14 (10–20)	15 (11–20)	15 (11–21)	17 (13–23)	20 (15–27)	16 (11–22)	<0.001
Alkaline Phosphatase (IU)	99 (77–130)	92 (74–119)	91 (72–116)	89 (71–111)	85 (69–106)	82 (68–102)	89 (71–113)	<0.001
Total Bilirubin (mg/dL)	0.5 (0.4–0.7)	0.5 (0.4–0.7)	0.6 (0.4–0.7)	0.6 (0.4–0.7)	0.6 (0.5–0.8)	0.7 (0.5–0.9)	0.6 (0.4–0.8)	<0.001
Gamma-glutamyltransferase (IU)	30 (18–63)	27 (18–51)	25 (16–42)	23 (17–39)	24 (18–38)	26 (19–40)	26 (18–44)	<0.001
Medication								
ACE-I/ARB/ARNI	751 (65)	849 (74)	879 (77)	915 (79)	916 (80)	917 (80)	5227 (76)	<0.001
Beta blockers	801 (70)	844 (74)	849 (74)	833 (72)	870 (76)	861 (75)	5058 (73)	0.02
Spironolactone	402 (35)	414 (36)	408 (36)	406 (35)	404 (35)	368 (32)	2402 (35)	0.39
Furosemide	852 (74)	879 (77)	868 (76)	774 (67)	670 (58)	486 (42)	4529 (66)	<0.001
Thiazide	102 (9)	131 (11)	146 (13)	185 (16)	183 (16)	152 (13)	899 (13)	<0.001
Digoxin	72 (6)	76 (7)	78 (7)	80 (7)	60 (5)	47 (4)	413 (6)	0.03
Amiodarone	200 (17)	224 (20)	177 (15)	185 (16)	169 (15)	119 (10)	1074 (16)	<0.001
Aspirin	543 (47)	590 (52)	580 (50)	624 (54)	666 (58)	733 (64)	3736 (54)	<0.001
Anti-Platelet	26 (2)	24 (2)	21 (2)	38 (3)	46 (4)	95 (8)	250 (4)	<0.001
New oral anticoagulants **	295 (26)	342 (30)	356 (31)	336 (29)	275 (24)	205 (18)	1809 (26)	<0.001
Vitamin K antagonists	171 (15)	202 (18)	149 (13)	147 (13)	164 (14)	140 (12)	973 (14)	0.002

Data is presented as median (inter-quartile range) for continuous variables and counts (percentages) for categorical variables. *p* value by the Kruskal–Wallis Test for continuous variables and the Chi-Square Test for categorical variables. Diabetes mellitus defined as fasting plasma glucose ≥ 126 mg/dL or glucose lowering treatment, hypertension as blood pressure > 140/90 mmHg measured on several occasions or anti-hypertensive treatment and hyperlipidemia as low density lipoprotein > 130 mg/dL, fasting serum triglycerides > 200 mg/dL or lipid lowering treatment. * Estimated glomerular filtration rate was calculated using the modified Modification of Diet in Renal Disease (MDRD) equation (175 * serum creatinine^–1.154^ * age^–0.203^. For females, a correction factor is used multiplying by 0.742.). ** Dabigatran, Rivaroxaban or Apixaban.

**Table 2:** Predictors of mortality by Cox regression analysis.

	Univariable		Multivariable	
	Hazard Ratio (95% CI)	*p* Value	Hazard Ratio (95% CI)	*p* Value
Age (years)	1.06 (1.05–1.06)	<0.001	1.04 (1.03–1.05)	<0.001
Gender (Male)	0.82 (0.74–0.90)	<0.001	1.17 (1.04–1.32)	0.010
NYHA III/IV	1.62 (1.53–1.72)	<0.001	1.45 (1.35–1.56)	<0.001
Diabetes Mellitus	1.16 (1.05–1.28)	0.003	1.18 (1.05–1.33)	0.006
Hypertension	2.36 (1.99–2.80)	<0.001	1.21 (0.98–1.49)	0.07
Ischemic Heart Disease	1.05 (0.95–1.17)	0.33	0.90 (0.80–1.02)	0.10
Atrial Fibrillation	1.61 (1.46–1.77)	<0.001	1.19 (1.06–1.33)	0.002
Body mass index * (kg/m^2^)	0.12 (0.07–0.22)	<0.001	0.16 (0.08–0.30)	<0.001
Urea (mg/dL) *	12.19 (9.89–15.02)	<0.001	4.25 (2.84–6.36)	<0.001
eGFR ** (mL/min per 1.73 m^2^)	0.82 (0.80–0.84)	<0.001	0.99 (0.95–1.04)	0.72
Sodium (mEq/L)	0.94 (0.92–0.95)	<0.001	0.95 (0.94–0.97)	<0.001
Hemoglobin RDW Ratio		<0.001		<0.001
Q1	6.03 (4.84–7.53)	<0.001	3.18 (2.43–4.16)	<0.001
Q2	4.47 (3.57–5.61)	<0.001	2.40 (1.84–3.15)	<0.001
Q3	3.39 (2.69–4.27)	<0.001	1.86 (1.42–2.45)	<0.001
Q4	2.67 (2.11–3.39)	<0.001	1.68 (1.27–2.21)	<0.001
Q5	1.68 (1.30–2.17)	<0.001	1.30 (0.97–1.74)	0.08
Q6	1.0 (Reference)		1.0 (Reference)	

Data is presented as hazard ratio (95% confidence interval), *p* value. * Log-transformed. ** Square root-transformed.

**Table 3:** Hazard ratio for clinical outcome according to hemoglobin RDW ratio by Cox regression analysis.

Hemoglobin RDW Ratio							
	Q1	Q2	Q3	Q4	Q5	Q6	*p* Value
Death							
Univariable	6.03 (4.84–7.53) <0.001	4.47 (3.57–5.61) <0.001	3.39 (2.69–4.27) <0.001	2.67 (2.11–3.39) <0.001	1.68 (1.30–2.17) <0.001	1.0 (Reference)	<0.001
Multivariable	3.18 (2.43–4.16) <0.001	2.40 (1.84–3.15) <0.001	1.86 (1.42–2.45) <0.001	1.68 (1.27–2.21) <0.001	1.30 (0.97–1.74) 0.08	1.0 (Reference)	<0.001
Multivariable and Drugs	3.11 (2.38–4.08) <0.001	2.50 (1.91–3.28) <0.001	1.97 (1.49–2.59) <0.001	1.76 (1.33–2.33) <0.001	1.35 (1.00–1.80) 0.05	1.0 (Reference)	<0.001
Death and cardiovascular hospitalization							
Univariable	2.72 (2.45–3.01) <0.001	2.02 (1.82–2.25) <0.001	1.58 (1.42–1.76) <0.001	1.49 (1.34–1.66) <0.001	1.22 (1.09–1.36) <0.001	1.0 (Reference)	<0.001
Multivariable	1.98 (1.76–2.23) <0.001	1.49 (1.32–1.68) <0.001	1.24 (1.10–1.39) <0.001	1.23 (1.09–1.39) <0.001	1.11 (0.98–1.25) 0.09	1.0 (Reference)	<0.001
Multivariable and Drugs	1.84 (1.63–2.08) <0.001	1.39 (1.23–1.57) <0.001	1.16 (1.03–1.30) 0.02	1.18 (1.05–1.33) 0.006	1.07 (0.95–1.21) 0.24	1.0 (Reference)	<0.001

Data is presented as hazard ratio (95% confidence interval), *p* value. Parameters that were included in the main multivariable analysis model were age, gender, NYHA functional class, diabetes, hypertension, ischemic heart disease, atrial fibrillation, log-transformed body mass index, log-transformed serum urea levels, square root-transformed estimated glomerular filtration rate, serum sodium and Hemoglobin RDW ratio. Parameters that were included in the multivariable and drugs analysis included the above parameters and the drug treatment with angiotensin-converting enzyme inhibitor/angiotensin receptor blocker/sacubitril-valsartan, beta blocker, furosemide and spironolactone.

## Data Availability

The data of this study are available from Clalit Health Service but restrictions apply to the availability of these data, which were used under the license for the current study, and are not publicly available.

## Supplementary Materials

The following supporting information can be downloaded at: https://www.mdpi.com/article/10.3390/jcm11030886/s1, Table S1. Hazard ratio for mortality in different cohorts of HF patients according to hemoglobin RDW ratio by Cox regression analysis.
