# Supplementary material for: The Ratio of Hemoglobin to Red Cell Distribution Width: A Strong Predictor of Clinical Outcome in Patients with Heart Failure
Source: J Clin Med. 2022 Feb 8;11(3):886. doi: 10.3390/jcm11030886 (PMC8836451; doi:10.3390/jcm11030886)
Supplement: Supplementary file 1 [file jcm-11-00886-s001.zip › jcm-1575769-supplementary.pdf]

Supplemental Table S1. Hazard ratio for mortality in different cohorts of HF patients according to hemoglobin RDW ratio by Cox regression analysis.

Hemoglobin RDW Ratio							
	Q1	Q2	Q3	Q4	Q5	Q6	P-value
HF reduced Ejection Fraction (N=1864)							
Univariable	5.84<br>(3.88-8.78)<br><0.001	4.34<br>(2.86-6.57)<br><0.001	2.75<br>(1.78-4.26)<br><0.001	1.87<br>(1.18-2.97)<br>0.008	1.22<br>(0.75-1.99)<br>0.42	1.0<br>(Reference)	<0.001
Multivariable	2.65<br>(1.62-4.32)<br><0.001	2.23<br>(1.37-3.63)<br>0.001	1.38<br>(0.83-2.29)<br>0.22	1.16<br>(0.69-1.95)<br>0.58	0.91<br>(0.53-1.57)<br>0.74	1.0<br>(Reference)	<0.001
Multivariable and Drugs	2.68<br>(1.62-4.42)<br><0.001	2.42<br>(1.47-3.96)<br><0.001	1.53<br>(0.91-2.56)<br>0.11	1.26<br>(0.75-2.13)<br>0.39	0.97<br>(0.56-1.68)<br>0.91	1.0<br>(Reference)	<0.001
HF preserved Ejection Fraction (N=2725)							
Univariable	5.52<br>(3.85-7.90)<br><0.001	3.92<br>(2.71-5.66)<br><0.001	3.45<br>(2.38-5.00)<br><0.001	2.81<br>(1.92-4.11)<br><0.001	1.93<br>(1.28-2.89)<br>0.002	1.0<br>(Reference)	<0.001
Multivariable	3.75<br>(2.43-5.78)<br><0.001	2.64<br>(1.70-4.10)<br><0.001	2.37<br>(1.53-3.69)<br><0.001	2.24<br>(1.44-3.51)<br><0.001	1.59<br>(0.99-2.55)<br>0.06	1.0<br>(Reference)	<0.001
Multivariable and Drugs	3.88<br>(2.51-6.00)<br><0.001	2.89<br>(1.86-4.50)<br><0.001	2.56<br>(1.64-3.99)<br><0.001	2.47<br>(1.57-3.86)<br><0.001	1.71<br>(1.07-2.75)<br>0.03	1.0<br>(Reference)	<0.001

HF Not-specified (N=2299)							
Univariable	5.97 (4.02-8.87) <0.001	4.87 (3.26-7.26) <0.001	3.26 (2.14-4.95) <0.001	2.88 (1.89-4.39) <0.001	1.70 (1.08-2.69) 0.02	1.0 (Reference)	<0.001
Multivariable	2.69 (1.64-4.41) <0.001	2.19 (1.34-3.58) 0.002	1.56 (0.94-2.58) 0.08	1.46 (0.88-2.42) 0.14	1.32 (0.78-2.23) 0.30	1.0 (Reference)	<0.001
Multivariable and Drugs	2.47 (1.50-4.06) <0.001	2.14 (1.31-3.49) 0.002	1.57 (0.95-2.61) 0.08	1.39 (0.84-2.32) 0.20	1.28 (0.76-2.17) 0.36	1.0 (Reference)	<0.001

Data is presented as hazard ratio (95% confidence interval), P value.

Parameters that were included in the main multivariable analysis model were age, gender, NYHA functional class, diabetes, hypertension, ischemic heart disease, atrial fibrillation, log-transformed body mass index, log-transformed serum urea levels, square root-transformed estimated glomerular filtration rate, serum sodium and Hemoglobin RDW ratio.

Parameters that were included in the multivariable and drugs analysis included the above parameters and the drug treatment with angiotensin-converting enzyme inhibitor/angiotensin receptor blocker/sacubitril-valsartan, beta blocker, furosemide and spironolactone.
